# Influence of Human Hunting Strategies and Large Carnivore Presence on Population Dynamics of European Facultative Scavengers

**DOI:** 10.1002/ece3.70424

**Published:** 2024-11-04

**Authors:** Elke Wenting, Jasper A. J. Eikelboom, Henk Siepel, Femke Broekhuis, Frank van Langevelde

**Affiliations:** ^1^ Department of Environmental Sciences Wageningen University and Research Wageningen The Netherlands; ^2^ Department of Ecology, Radboud Institute for Biological and Environmental Sciences Radboud University Nijmegen The Netherlands

**Keywords:** Lotka‐Volterra, population modelling, predator–prey dynamics, ungulates

## Abstract

Ungulates serve as the primary carrion source for facultative scavengers in European ecosystems. In the absence of large carnivores, such as wolves (*Canis lupus*), human hunting leftovers are the main source of carrion for these scavengers. Additionally, wild boars (*Sus scrofa*) are heavily culled in many ecosystems and are both a significant prey species for wolves as well as a key scavenger. Nowadays, wolves and wild boars are re‐establishing their historical home ranges. However, it remains unclear how their presence influences the population dynamics of facultative scavengers under different scenarios of human hunting strategies. We simulated the biomass densities of all states in the trophic web including European scavengers and wolves using an ordinary differential equations (ODE) model. The presence of wolves led to a positive trend in scavenger biomass in general. However, in general, we found that plant‐based resources were more important for scavenger dynamics than carrion, regardless of whether the carrion originated from human hunting or wolf predation. Only when wolves were absent but boars present, the human hunting strategy became important in determining scavenger dynamics via carrion supply. In conclusion, our model indicates that population dynamics of facultative scavengers are not mainly driven by the availability of carrion, but rather by the presence of and competition for vegetation. Furthermore, our simulations highlight the importance of adapting human hunting strategies in accordance with the re‐establishment of wolf and boar as these can cause fluctuating population patterns over the years.

## Graphical abstract

Ungulates are the major source of carrion in European ecosystems, with human hunting leftovers being important in the absence of large carnivores like wolves. However, although our model shows that carrion is not the major food source for facultative scavengers, carrion can be important for these scavengers under some conditions.

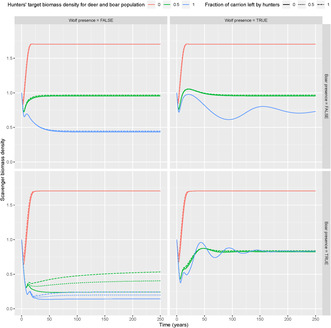

## 1 Introduction

The decomposition of dead animal bodies – carrion – is an important ecological process that can have far‐reaching consequences for ecosystem functioning (Wenting et al. 2023, 2024). Most of the carrion in terrestrial ecosystems is consumed by scavengers (DeVault, Rhodes Jr, and Shivik 2003; Wilson and Wolkovich 2011). The major source of carrion in many ecosystems, including European temperate woodlands, consists of large ungulates (Beasley et al. 2019; Moleón et al. 2019; Greenspoon et al. 2023). This includes species like red deer (*Cervus elaphus*), fallow deer (*Dama dama*), and wild boar (*Sus scrofa*). Anthropogenic hunting is one of the major causes of death of free roaming ungulates, especially in areas where large carnivores no longer occur due to extermination (Gordon 2009; Found 2016; Williams et al. 2017).

Currently, however, populations of large carnivores are re‐establishing to their historical ranges across Europe (Chapron et al. 2014; Galaverni et al. 2016). An example is the grey wolf (*Canis lupus*), a social apex predator with large dispersal rates and large territories (Jędrzejewski et al. 2007), that expanded its distribution extensively over the past decades (Planillo et al. 2023). The re‐establishment of the wolf has been possible due to strict legal protection and the recovery of large herbivore populations (Chapron et al. 2014). The presence of the wolf can have cascading effects on ecosystem functioning (Allen et al. 2017), for example by indirectly changing the diet of grizzly bears (*Ursus arctos horribilis*) to more plant‐based (Ripple et al. 2015) and willow recovery through behavioural changes of herbivores (Marshall, Cooper, and Hobbs 2014). This is well‐studied in North American wolf habitats (Lesmerises, Dussault, and St‐Laurent 2012; Ripple and Beschta 2012; Ford and Goheen 2015; Gantchoff et al. 2022). The European situation is considerably less well‐studied (Nowak et al. 2017; Reinhardt et al. 2019), despite that there are essential differences between the European and North American continent. Since it is generally harder to predict trophic cascades in more human‐dominated landscapes such as European ecosystems (Hebblewhite et al. 2005; Muhly et al. 2013; Dorresteijn et al. 2015), insights obtained from North American wolf habitats might not be equally relevant in European wolf habitats (Focardi et al. 2017).

One of the most notable differences between European and American ecosystems is the importance of wild boar as both abundant ungulate, scavenger species, and prey species for wolves (Focardi et al. 2017). The wild boar is a wide‐spread non‐ruminant ungulate that is widely described as an ecosystem engineer due to its extensive rooting behaviour (Sandom, Hughes, and Macdonald 2013; Ballari and Barrios‐García 2014; Baruzzi and Krofel 2017; Barrios‐Garcia et al. 2023). It is a well‐known scavenger species (Selva et al. 2005; Selva and Fortuna 2007; Focardi et al. 2008) that can contribute considerably to carrion removal from ecosystems (Wenting, Rinzema, and van Langevelde 2022; Wenting et al. 2024; Newsome et al. 2023). Although wild boars are not tolerated by humans everywhere in Europe (Boonman‐Berson, Driessen, and Turnhout 2019), hence not everywhere present as prey species, they are reported as a noticeable part of the wolves' diet throughout European ecosystems in areas where they occur (Smietana and Klimek 1993; Ansorge, Kluth, and Hahne 2006; Nores, Llaneza, and Álvarez 2008; Lanszki et al. 2012; Špinkytė‐Bačkaitienė and Pėtelis 2012; Barja et al. 2023). That implies that the wild boar is an important prey species for wolves (Mattioli et al. 2011; Mori et al. 2017) and also an important scavenger in wolf habitats (Focardi et al. 2017).

The presence of large carnivores like wolves can influence the process of scavenging in ecosystems. Through only partially consuming their prey, wolves can indirectly facilitate scavengers (Vucetich, Vucetich, and Peterson 2012; Focardi et al. 2017; Boczulak et al. 2023). Wolves might facilitate consumption efficiency of vultures, corvids and smaller mammals by tearing open thick‐skinned carcasses (Moleón et al. 2014). Partial prey consumption is common behaviour for wolves, being the combined result of pack size, prey size, and completeness of consumption in first sitting (Sand et al. 2012; Vucetich, Vucetich, and Peterson 2012; Mech and Boitani 2019). In North America, it has been described that common ravens (*Corvus corax*) use activity patterns of wolves to benefit from wolf kills, as a feeding strategy in winter (Stahler, Heinrich, and Smith 2002; Walker et al. 2018). However, scavenger dynamics might not change in the same way in different systems because scavenger species adapt their behaviour based on the local circumstances. Klauder et al. (2021), for instance, found that red foxes (*Vulpes vulpes*) were least likely to visit wolf kills in Denali National Park and Preserve, Alaska. This contradicts to findings in Europe and elsewhere in North America, where red foxes are reported to visit up to 90% of wolf‐predated ungulates (Selva 2004; Wikenros, Ståhlberg, and Sand 2014; O'Malley et al. 2018). Thus, the potential impact of re‐establishing wolf populations on scavenger dynamics can be system specific (Laundré, Hernández, and Altendorf 2001; Levi and Wilmers 2012; Haswell, Kusak, and Hayward 2017; Kuijper et al. 2024), increasing the need to investigate potential influences of re‐establishing wolves under different circumstances.

It has been described that different causes of death of ungulates – e.g., originated from human hunting or predated by wolves – can differently influence scavengers. For instance, predator‐kills were mostly preferred by scavengers in the Białowieża Primaeval Forest, Poland (Selva 2004; Selva and Fortuna 2007). Carrion obtained from human hunting can also facilitate a wide range of scavenger species (Mateo‐Tomás et al. 2015), in some cases even more than wolf kills (Ho et al. 2023). It remains unclear to which extent such differences might be due to different human hunting strategies, e.g., hunting target (‘pressure’) or the fraction of carrion left for scavengers. Also, the actual importance of carrion versus other resources for facultative scavengers – that frequently consume but do not depend on carrion (Wilson and Wolkovich 2011) – remains unclear.

Thus, summarising, it remains unclear how human hunting strategies and the presence of wolves and/or wild boar (henceforth ‘boar’) influence the population dynamics of European facultative scavengers (henceforth ‘scavengers’). We focus on (vertebrate) species that consume plant‐based food and carrion primarily and are flexible in their diet and behaviour (Selva and Fortuna 2007; Wenting, Rinzema, and van Langevelde 2022; Wenting et al. 2024). These include corvids like common raven and carrion crow (*Corvus corone*), and mesocarnivores, for instance red fox, European badger (*Melis melis*), raccoon (*Procyon lotor*) and other mustelids including beach marten (*Martes foina*), pine marten (*Martes martes*) and European polecat (*Mustela putorius*) (Díaz‐Ruiz et al. 2013; Rooney and Montgomery 2013; Papakosta et al. 2014; Libois et al. 2019; Jain et al. 2022). In this study, we use a differential‐equations modelling approach to examine how different human hunting strategies combined with the presence or absence of wolf and boar influence the population dynamics of scavengers. We address two research questions: (1) What is the influence of human hunting strategies in interaction with the presence or absence of boar and wolf, on scavenger population dynamics? and (2) What is the relative importance of carrion for scavenger population dynamics under different human hunting strategies in interaction with the presence or absence of boar and wolf?

## 2 Methods

### 2.1 Model Description and Assumptions

**FIGURE 1:**
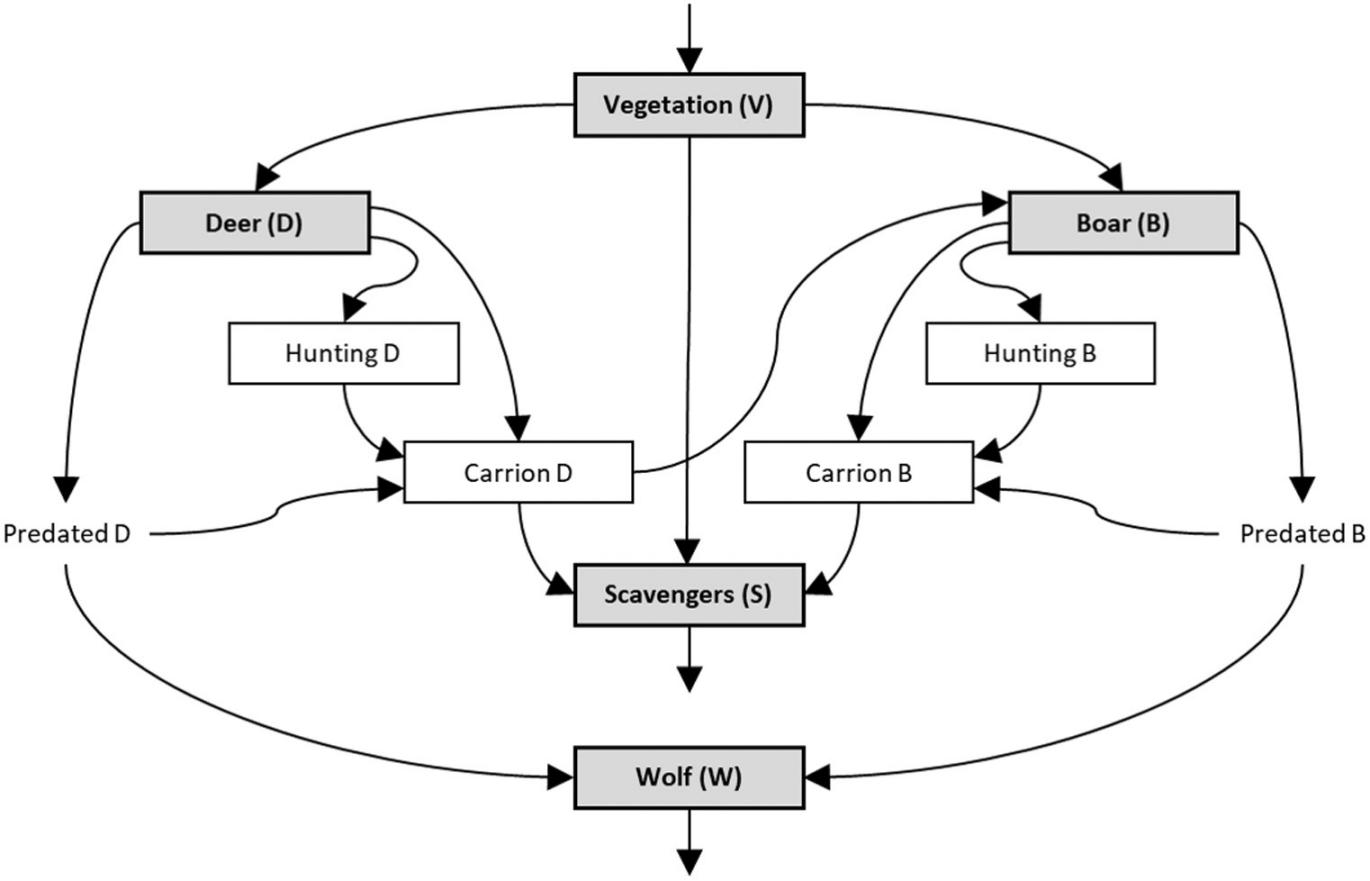
Trophic web of European scavengers and wolves. The consumers of vegetation (V) consist of two types of ungulates – deer (D) and boar (B) – and facultative scavengers (S). V represents vegetation and all other resources, including small prey of facultative scavengers, combined. The D species represent all Cervidae species, whereas the B represents wild boar (*Sus scrofa*). The S species represent all facultative scavengers, including vertebrates and invertebrates. Both D and B populations can be hunted, e.g., regular culling practices by humans. S species consume both D and B carrion, whereas B only scavenge on D carrion, i.e., we assume no cannibalism. Carrion from D is first consumed by B, then S. The large predator wolf (W) predates both on D and B, of which a fraction enters the carrion pool and is thus not consumed by W.

We simulated the biomass densities of a trophic web of European scavengers and wolves (Figure 1) using an ordinary differential equations (ODE) model. We based our model on the model developed by Focardi et al. (2017) for scavenger/predator systems, but changed three main things: (1) we added a separate state for scavengers, (2) implemented human hunting on boar and deer, and (3) merged adult boar and piglets into one state to simplify the model. The other details of the model by Focardi et al. (2017) are similar to our model specifications that we explain here. In our model, vegetation V is consumed by deer D, boar B and scavengers S (Equation 1), which we further subdivided here into Equations (1a)–(1d). Here, vegetation includes all plant‐based materials. Deer are consumed by wolf W, killed by hunters and die of other causes (Equation 2, subdivided over Equations (2a), (2b), (2c), (2d)). For boar the same applies as for deer, but they also consume deer carrion instead of only vegetation (Equation 3, subdivided over Equations (3a), (3b), (3c), (3d), (3e)). Scavengers consume both vegetation and the carrion from deer and boar, and die of natural causes (Equation 4, subdivided over Equations (4a), (4b), (4c), (4d)). Wolves thus consume deer and boar and die of natural causes (Equation 5, subdivided over Equations (5a), (5b), (5c)). For simplicity, we assumed no scavenging behaviour by wolves, nor did we assume that scavengers consume wolf carrion (as wolf carrion only makes up a small portion of the total amount of carrion).

(1) $$\frac{\mathrm{dV}}{\mathrm{dt}}=V_{\text{growth}}-V_{\text{consD}}-V_{\text{consB}}-V_{\text{consS}}$$

(2) $$\begin{array}{c} \frac{\mathrm{dD}}{\mathrm{dt}}=D_{\text{growthV}}-D_{\text{pred}}-D_{\text{hunt}}-D_{\text{death}} \end{array}$$

(3) $$\begin{array}{c} \frac{\mathrm{dB}}{\mathrm{dt}}=B_{\text{growthV}}+B_{\text{growthD}}-B_{\text{pred}}-B_{\text{hunt}}-B_{\text{death}} \end{array}$$

(4) $$\begin{array}{c} \frac{\mathrm{dS}}{\mathrm{dt}}=S_{\text{growthV}}+S_{\text{growthD}}+S_{\text{growthB}}-S_{\text{death}} \end{array}$$

(5) $$\begin{array}{c} \frac{\mathrm{dW}}{\mathrm{dt}}=W_{\text{growthD}}+W_{\text{growthB}}-W_{\text{death}} \end{array}$$

A description of all variables and the references underlying their parameter estimations are presented in Table 1, where in the text we only elaborate on the variables that are needed to understand the working of the equations. All vegetation biomass is modelled in one state and follows the regrowth equation of Turchin and Batzli (2001), where biomass is expressed in normalised values with respect to the carrying capacity $k_{0}$ and grows with rate $R_{0}$ (Equation 1a).

(1a) $$\begin{array}{c} V_{\text{growth}}=R_{0}V\left(1-\frac{V}{k_{0}}\right)\; \end{array}$$

**TABLE 1:** Parameter values.

Symbol	Meaning	Initial value	Value after sensitivity analysis	Unit	Reference/notes
*R*_0_	Regrowth rate of V	4	4	year^−1^	Focardi et al. (2017)
*k*_0_	Carrying capacity of V	10	10	ton ha^−1^	Normalised vegetation biomass density (as in Focardi et al. (2017)), unit is an approximation using Earth's total plant biomass (Bar‐On, Phillips, and Milo 2018)
*A*_*VD*_	Ingestion of resources by D per unit D for overwhelming V	23.5	23.5	year^−1^	Based on individual food requirement of 986 kg year^−1^ (Mulley 2002) and average individual weight of 42 kg (Moore, Littlejohn, and Cowie 1988)
*B*_*XX*_	Half‐saturation density of resources in the foraging of D, B, S and W	10	10	ton ha^−1^	Based on Focardi et al. (2017)
*C*_*VD*_	Conversion factor from consumed resource V to D	0.017	0.025	—	Based on Flajšman, Jerina, and Pokorny (2017), Mulley (2002), and Moore, Littlejohn, and Cowie (1988), see conversion coefficient calculations (Appendix S1)
*M*_*D*_	Death rate of D in the absence of hunting or predators	0.125	0.125	year^−1^	Based on Müller et al. (2010); mean life expectancy in captivity is ~8 years
*A*_*XB*_	Ingestion of resources by B per unit B for overwhelming total resources and average carrion in diet	20.15	20.15	year^−1^	Based on individual food requirement of 1209 kg year^−1^ (Nagy 2021; Treyer et al. 2012) and assumed average individual weight of 60 kg with 16% carrion in diet
*C*_*VB*_	Conversion factor from consumed resource V to B	0.055	0.055	—	Based on Chinn et al. (2022), Gethöffer, Sodeikat, and Pohlmeyer (2007), Treyer et al. (2012), Sá, Moreno, and Carciofi (2020), see conversion coefficient calculations (Appendix S1)
*M*_*B*_	Death rate of B in the absence of hunting or predators	0.08	0.08	year^−1^	Based on Massei (1995): maximum age of 12 years
*u*_*a*_	Average portion of D carrion scavenged by B	0.16	0.16	—	Ballari and Barrios‐García (2014)
*u*	Maximum portion of D carrion scavenged by B	0.2	0.2	—	Unknown, tested during sensitivity analyses and estimated based on average 16% of Ballari and Barrios‐García (2014)
*B*_*u*_	Half‐saturation density of carrion portion in the scavenging of B on D carrion	0.1	0.1	—	Unknown, tested during sensitivity analyses and estimated based on average 16% of Ballari and Barrios‐García (2014)
*C*_*DB*_	Conversion factor from consumed resource D to B	0.069	0.069	—	Based on Chinn et al. (2022), Gethöffer, Sodeikat, and Pohlmeyer (2007), Treyer et al. (2012), see conversion coefficient calculations (Appendix S1)
*q*	Exponential decay rate of natural death rate of deer and boar with increasing predator density	10	10	—	Unknown, tested during sensitivity analyses
*T*_*D*_	Targeted population density for D by hunters	0–1	0–1	ton ha^−1^	Varied to analyse effect of hunting regimes
*H*_*D*_	Hunting rate of D above aimpopD at overwhelming D	0–1	0–1	year^−1^	Varied to analyse effect of hunting regimes
*L*_*D*_	Not harvested portion of hunted D	0–1	0–1	—	Varied to analyse effect of hunting regimes
*T*_*B*_	Targeted population density for B by hunters	0–1	0–1	ton ha^−1^	Varied to analyse effect of hunting regimes
*H*_*B*_	Hunting rate of B above aimpopB at overwhelming B	0–1	0–1	year^−1^	Varied to analyse effect of hunting regimes
*L*_*B*_	Not harvested portion of hunted B	0–1	0–1	—	Varied to analyse effect of hunting regimes
*M*_*S*_	Death rate of S	0.2	0.2	year^−1^	Based on overall death rate as in Focardi et al. (2017)
*C*_*XS*_	Conversion factor from consumed resource D or B to S	0.054	0.1	—	Mean animal based conversion factor
*M*_*W*_	Death rate of W	0.07	0.2	year^−1^	Based on Hannon and Ruth (2001); life expectancy in captivity is max. 14 years
*r*	Predation rate by W per unit W for overwhelming resources	96.6	96.6	year^−1^	Based on individual food requirement of 1642.5 kg year^−1^ (Jędrzejewski et al. 2002) and average individual weight of 25 kg (Jędrzejewski et al. 2002) and corrected for unconsumed portion
*v*	Portion of predated resources by W not consumed by W	0.32	0.32	—	Based on Metz et al. (2011) and Wilmers, Crabtree, et al. (2003)
*C*_*XW*_	Conversion factor from consumed resource to W	0.038	0.038	—	Based on Jędrzejewski et al. (2002), Sidorovich et al. (2007), see conversion coefficient calculations (Appendix S1)
*A*_*XS*_	Ingestion of resources by S per unit S for overwhelming resources	20	20	year^−1^	Unknown, tested during sensitivity analyses
*C*_*VS*_	Conversion factor from consumed resource V to S	0.036	0.036	—	Mean plant based conversion factor

*Note:* See Appendix S1 for the conversion factor calculations.

The consumption rates of vegetation by deer (Equation 1b), boar (Equation 1c) and scavengers (Equation 1d) all follow a Holling type II functional response (Holling 1966), which is often used to describe the realistic ‘levelling‐off’ of a response with increasing resources (Skalski and Gilliam 2001).

(1b) $$\begin{array}{c} V_{\text{consD}}=D\frac{A_{\mathrm{VD}}V}{B_{\mathrm{XX}}+V} \end{array}$$

(1c) $$\begin{array}{c} V_{\text{consB}}=B{\dddot{A}}_{\mathrm{XB}}\left(1-\ddot{u}\right) \end{array}$$

(1d) $$\begin{array}{c} V_{\text{consS}}=S\frac{{\ddot{A}}_{\mathrm{XS}}V}{\left(K_{D}-{\dddot{A}}_{\mathrm{XB}}\ddot{u}B\right)+K_{B}+V} \end{array}$$

$A_{\mathrm{YZ}}$ is the maximum amount of resources Y ingested per unit Z (e.g., $A_{\mathrm{VD}}$ is the maximum amount of vegetation ingested per unit of deer), with $B_{\mathrm{YZ}}$ being the half‐saturation density of Y per unit Z (which we kept at the same value $B_{\mathrm{XX}}$ (Table 1) for all functional response equations in our model, as these values are very difficult to estimate (Skalski and Gilliam 2001)) to determine the actual ingestion rate via this functional response.

Equation (1b) has its functional response written in its most basic form, given that deer only consume vegetation in our model. However, boar (Equation 1c) also consume deer carrion $K_{D}$, and scavengers (Equation 1d) also consume both deer and boar $K_{B}$ carrion, which influences their vegetation consumption rate per time step. Therefore, we extended upon the default Holling type II functional response equations of the vegetation consumption by boar and scavengers, which we list here as separate equations to be substituted in the main equations. For example for boar, we model the portion of deer carrion in their diet $\ddot{u}$ with a separate Holling type II functional response (Equation 1c2), based on the amount of available deer carrion.

(1c2) $$\begin{array}{c} \ddot{u}=\frac{u\frac{K_{D}}{A_{\mathrm{XB}}B}}{B_{u}+\frac{K_{D}}{A_{\mathrm{XB}}B}} \end{array}$$

Given that carrion is more nutritious than vegetation for boar, the conversion factor from a unit consumed vegetation biomass to a unit boar $C_{\mathrm{VB}}$ is smaller than the conversion factor from deer carrion to boar $C_{\mathrm{DB}}$ (Table 1; Appendix S1). As such, we model the maximum total consumption rate by boar ${\ddot{A}}_{\mathrm{XB}}$ so that it consumes less biomass, when more of its diet consists of carrion (Equation 1c1a).

(1c1a) $$\begin{array}{c} {\ddot{A}}_{\mathrm{XB}}=A_{\mathrm{XB}}\frac{C_{\mathrm{VB}}\left(1-u_{a}\right)+C_{\mathrm{DB}}u_{a}}{C_{\mathrm{VB}}\left(1-\ddot{u}\right)+C_{\mathrm{DB}}\ddot{u}} \end{array}$$

This way a unit of boar ‘aims to’ obtain approximately the same amount of boar biomass units in total ${\dddot{A}}_{\mathrm{XB}}$ via a Holling type II functional response (Equation 1c1), independent of the fraction of carrion in its diet.

(1c1) $$\begin{array}{c} {\dddot{A}}_{\mathrm{XB}}=\frac{{\ddot{A}}_{\mathrm{XB}}\left(V\left(1-\ddot{u}\right)+K_{D}\ddot{u}\right)}{B_{\mathrm{XX}}+V\left(1-\ddot{u}\right)+K_{D}\ddot{u}} \end{array}$$

For scavengers, their maximum total consumption rate ${\ddot{A}}_{\mathrm{XS}}$ is also computed via a Holling type II functional response (Equation 1d1), which considers the available vegetation, deer carrion and boar carrion biomass.

(1d1) $$\begin{array}{c} {\ddot{A}}_{\mathrm{XS}}=\frac{A_{\mathrm{XS}}\left(V+\left(K_{D}-{\dddot{A}}_{\mathrm{XB}}\ddot{u}B\right)+K_{B}\right)}{B_{\mathrm{XX}}+V+\left(K_{D}-{\dddot{A}}_{\mathrm{XB}}\ddot{u}B\right)+K_{B}} \end{array}$$

Given that we assume boars are the first and foremost scavengers to consume deer carrion (Wenting, Rinzema, and van Langevelde 2022; Wenting et al. 2024), only the deer carrion that is not consumed by boar are available for other scavengers. Deer (Equation 1d2) and boar carrion (Equation 1d3) are (i) produced by natural mortality, (ii) the fraction that is left by human hunters and (iii) the fraction that is left by wolves.

(1d2) $$K_{D}=D_{\mathrm{death}}+\;D_{\mathrm{hunt}}L_{D}\;+\;D_{\mathrm{pred}}v$$

(1d3) $$\begin{array}{c} K_{B}=B_{\text{death}}+B_{\text{hunt}}L_{B}+B_{\text{pred}}v \end{array}$$

The functions that describe the growth of deer (Equation 2a), boar (Equation 3a) and scavengers (Equation 4a) from vegetation are all calculated by multiplying the consumed vegetation biomass by the conversion factor $C_{\mathrm{VY}}$ from a unit consumed vegetation biomass to a unit Y.

(2a) $$\begin{array}{c} D_{\text{growthV}}=V_{\text{consD}}C_{\mathrm{VD}}\; \end{array}$$

(3a) $$\begin{array}{c} B_{\text{growthV}}=V_{\text{consB}}C_{\mathrm{VB}}\; \end{array}$$

(4a) $$\begin{array}{c} S_{\text{growthV}}=V_{\text{consS}}C_{\mathrm{VS}}\; \end{array}$$

The deer carrion growth function of boar (Equation 3b) is obtained by multiplying the portion of deer carrion in the boars' diet $\ddot{u}$ by the total consumed biomass per unit boar ${\dddot{A}}_{\mathrm{XB}}$, the deer carrion to boar conversion factor $C_{\mathrm{DB}}$ and the total units of boar.

(3b) $$\begin{array}{c} B_{\text{growthD}}={\dddot{A}}_{\mathrm{XB}}\ddot{u}BC_{\mathrm{DB}}\; \end{array}$$

The deer (Equation 4b) and boar carrion (Equation 4c) growth functions of scavengers are also obtained by multiplying the consumed carrion biomass by the carrion to scavenger conversion factor $C_{\mathrm{XS}}$.

(4b) $$\begin{array}{c} S_{\text{growthD}}=\min\left[\left({\ddot{A}}_{\mathrm{XS}}S\right),\left(K_{D}-{\dddot{A}}_{\mathrm{XB}}\ddot{u}B\right)\right]C_{\mathrm{XS}}\; \end{array}$$

(4c) $$\begin{array}{c} S_{\text{growthB}}=\min\left[\left({\ddot{A}}_{\mathrm{XS}}S\right),\left(K_{B}\right)\right]C_{\mathrm{XS}}\; \end{array}$$

The consumed carrion biomass by scavengers is modelled with a Holling type I functional response (Holling 1966), meaning that scavengers will consume ${\ddot{A}}_{\mathrm{XS}}$ per unit S until a maximum value that is equal to the total amount of available carrion. However, do note that ${\ddot{A}}_{\mathrm{XS}}$ itself is computed via a Holling type II functional response (Equation 1d1), so the overall carrion consumption by and subsequent growth of scavengers follows a Holling type II functional response in relation to resource availability.

The deer (Equation 5a) and boar growth functions of wolf (Equation 5b) are also similar in structure as the other growth functions, where the amount of predated deer $D_{\text{pred}}$ and boar $B_{\text{pred}}$ (both explained in the next paragraph) are multiplied by the conversion factor $C_{\mathrm{XW}}$ and multiplied by the fraction of the carrion that is not left behind by the wolves $\left(1-v\right)$.

(5a) $$W_{\text{growthD}}=D_{\text{pred}}C_{\mathrm{XW}}\left(1-v\right)$$

(5b) $$\begin{array}{c} W_{\text{growthB}}=B_{\text{pred}}C_{\mathrm{XW}}\left(1-v\right)\; \end{array}$$

The predation of deer (Equation 2b) and boar by wolves (Equation 3c) are both also modelled with a Holling type II functional response, where r is the maximum total predation rate per wolf unit. The wolves' total predation rate is divided over deer and boar based on their relative availability. We amplified this selection preference of wolf for the most abundant prey by squaring the deer and boar biomass densities, so that it was easier to simulate a system in which both deer and boar could co‐occur despite the higher vegetation conversion factors of boar versus deer (Table 1). This way we assumed that wolves became more specialistic hunters for a single prey species when that species was abundant compared to the other species (Becker et al. 2008; Sand et al. 2016; Zabihi‐Seissan, Prokopenko, and Vander Wal 2022).

(2b) $$\begin{array}{c} D_{\text{pred}}=\frac{r\left(D+B\right)}{B_{\mathrm{XX}}+D+B}\frac{D^{2}}{D^{2}+B^{2}}W\; \end{array}$$

(3c) $$\begin{array}{c} B_{\text{pred}}=\frac{r\left(D+B\right)}{B_{\mathrm{XX}}+D+B}\frac{B^{2}}{D^{2}+B^{2}}W\; \end{array}$$

Hunting of both deer (Equation 2c) and boar (Equation 3d) is zero when their biomass is equal or below the hunters' target biomass T. When their biomass is higher, then only the amount above this target biomass is hunted with a hunting efficiency rate H (to simulate the increasing difficulty to find animals to hunt when their density drops). This describes hunting regimes that are standard in European countries, where the hunting quota of animals are determined based on the yearly estimated population size and the target population size, but where quota are often not fully realised when these targets are strict (Dijkhuis et al. 2023).

(2c) $$D_{\text{hunt}}=\left\{\begin{array}{rr} 0, & \text{if}\;D\leq T_{D}\\ \left(1-\frac{T_{D}}{D}\right)H_{D}\left(D-T_{D}\right), & \text{if}\;D>T_{D} \end{array}\right.$$

(3d) $$B_{\text{hunt}}=\left\{\begin{array}{rr} 0, & \text{if}\;B\leq T_{B}\\ \left(1-\frac{T_{B}}{B}\right)H_{B}\left(B-T_{B}\right), & \text{if}\;B>T_{B} \end{array}\right.$$

Finally, the natural mortality of deer (Equation 2d), boar (Equation 3e), scavengers (Equation 4d) and wolves (Equation 5c) are modelled by multiplying a static death rate M with the total biomass units of the respective populations. For both deer and boar, this natural mortality decreases with an exponential decay rate of q multiplied by the wolves' predation pressure. We implemented this process to simulate that wolves more often target old and weak prey, thereby lowering the natural mortality rate of these prey animals (Becker et al. 2008; Kittle et al. 2017).

(2d) $$\begin{array}{c} D_{\text{death}}=e^{-\frac{\mathrm{qW}D^{2}}{D\left(D^{2}+B^{2}\right)}}M_{D}D\; \end{array}$$

(3e) $$\begin{array}{c} B_{\text{death}}=e^{-\frac{\mathrm{qW}B^{2}}{B\left(D^{2}+B^{2}\right)}}M_{B}B\; \end{array}$$

(4d) $$\begin{array}{c} S_{\text{death}}=M_{S}S\; \end{array}$$

(5c) $$\begin{array}{c} W_{\text{death}}=M_{W}W\; \end{array}$$

### 2.2 Parameter Estimation and Sensitivity Analysis

We aimed to develop an ODE model that resembles the actual processes of a temperate ecosystem, which is a non‐trivial task. Especially the estimation of parameter values is not straightforward, because (1) not all parameter values can be estimated directly from the literature and (2) even parameter values derived from the literature may cause non‐realistic simulations, given the simplifications of a model compared to reality. We approached this problem with a three‐step workflow. First, we searched the literature using keyword based on the explained meaning of the parameters (Table 1) to estimate the parameter values. Second, we built up the complexity our model step‐by‐step (first a model only with vegetation (by setting the initial values of all other states at zero), then vegetation + deer, then vegetation + boar, etc.; see R script via link in Data Accessibility Statement), to estimate the values of the other parameters and to finetune the parameters that we based on the literature. These values were estimated to avoid both chaotic time series and crashing populations, when these were unrealistic patterns for the simulated scenarios based on our expert knowledge. When we needed to update parameter values, we updated them such that it would strike a balance between changing as few parameters as possible with as small a deviation per parameter as possible (Table 1). Third, during each step of this workflow, we also performed sensitivity analyses on the parameters to check that the simulations were relatively robust to alterations of our estimated parameter values (see R script via link in Data Accessibility Statement). At each step of this workflow, we varied the parameters that were introduced at this step by a factor of 0.75, 0.875, 1, 1.125 and 1.25. Then we ran the simulations for all combinations of these parameter values at each step of our workflow (e.g., so $5^{4}=625$ simulations in a single step when 4 parameters were introduced). Then we examined the output of the simulations using: (1) timeseries line charts of the different states (e.g., V) with multiple lines and figure panels for the different parameter values of the sensitivity analysis and (2) 2D image plots of the end state of the different states (e.g., V) with two parameters that were varied during the sensitivity analysis along both the *x*‐ and *y*‐axis of the image plots and the other varied parameter values separated over multiple figure panels. When the qualitative patterns of the simulations were highly dependent on the parameter value range that we chose during our sensitivity analyses, then we updated our estimated parameter values in the same way as in step two to make the simulations more robust. Finally, at the end of each step, we visualised phase planes of each combination of two states to verify if the initial state values influenced the end states (which was never the case, i.e., all models converged to a single stable state).

After this three‐step workflow to estimate parameter values was complete, we let our simulation run with these same parameter values for four different scenarios: with and without both boar and wolf (i.e., wolf and boar, only wolf, only boar, neither), by iteratively setting the initial state value of boar and wolf at zero. For each of these four scenarios, we also varied two parameters of interest: (1) the hunters' target biomass for both the deer and population and (2) the fraction of carrion left by hunters. Finally, when our interpretations of the results were highly dependent on a single parameter value, we performed a sensitivity analysis for this parameter at this stage again to test the robustness of our conclusions.

### 2.3 Numerical Simulations

We performed the numerical simulations in *R 4*.*3*.*1* (R Core Team 2023) with the *deSolve* package to solve the ODE model (Soetaert, Petzoldt, and Setzer 2010), the *data*. *table* package to process the data (Dowle and Srinivasan 2023), and the *ggplot2* package to visualise (Wickham 2016). We used *lsoda* as the ODE solving algorithm (Petzold 1983), which switches automatically between stiff and non‐stiff methods. As such, this algorithm adaptively changes the time step size during integration to e.g., avoid overshooting. We let the simulations of all our different scenarios run for 250 time‐steps (years), because this was long enough to stabilise the different states from its initial values and still short enough to visually investigate the evolution of the states over time.

## 3 Results

### 3.1 Effect of Wild Boar on Scavenger Dynamics

**FIGURE 2:**
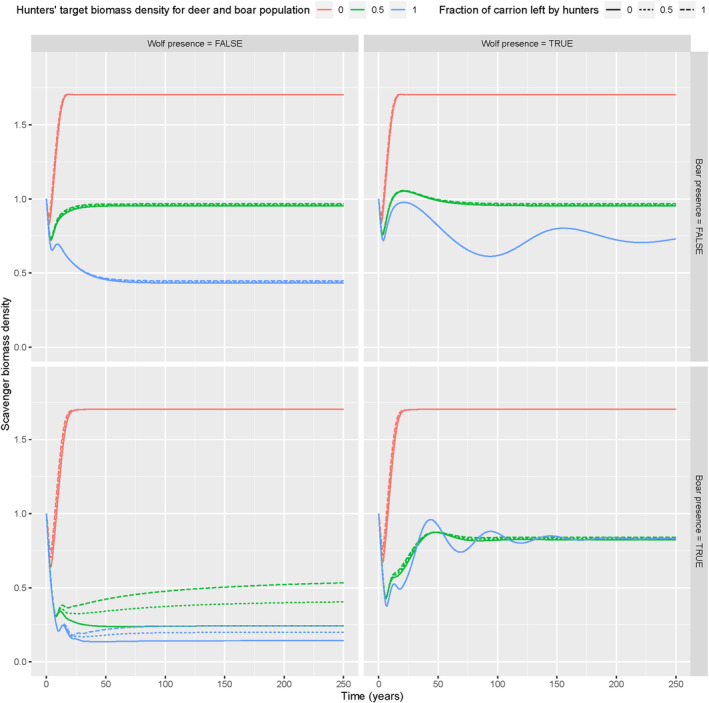
Scavenger biomass density ODE model simulations (*y*‐axis) over time (*x*‐axis), with boar (horizontal panels) and wolf present/absent (vertical panels), for different hunting target values (line colours) and fractions of carrion left by hunters (line types).

In the scenarios with a population target of 0, i.e., more hunting, all deer and boar became extinct (Appendix S2: Figures S2.1– S2.2), so, to assess the effect of boar on scavenger dynamics, we focused on the scenarios with a high or medium hunting target (Figure 2). When boar is present but wolf absent, we observed that the overall scavenger biomass was the lowest (Figure 2). In this scenario, there is more competition for vegetation resources between boar, deer and scavengers (Appendix S2: Figures S2.1–S2.3). Deer biomass is higher in the absence of boar (Appendix S2: Figure S2.1), but in the presence of boar, there is more biomass of deer and boar combined (Appendix S2: Figures S2.1 and S2.2). This means that competition for vegetation resources would drive scavenger biomass, rather than competition for carrion. This becomes also apparent from the lower vegetation biomass in the scenario with boar and without wolves (Appendix S2: Figure S2.3).

The importance of vegetation resources in determining scavenger biomass could be heavily influenced by the parameter value we used for the conversion factor of vegetation for scavengers $C_{\mathrm{VS}}$. We assessed the importance of this parameter value with a sensitivity analysis. When $C_{\mathrm{VS}}$ was 30% higher, the same qualitative time series patterns of scavenger biomass occurred for all scenarios, with only the absolute scavenger biomass values becoming higher by a factor of 1–1.25 (Appendix S3: Figures S3.2 and S3.3). Similarly, we found the same patterns, but with lower absolute biomass values by a factor of 0.5–1, when $C_{\mathrm{VS}}$ was 30% lower (Appendix S3: Figures S3.1 and S3.2). That means that our results are robust to varying values of the conversion factor of vegetation for scavengers. Thus, the observation that vegetation resources, rather than carrion, are limiting scavenger biomass is robust. Our simulations showed that the effect of boar on scavenger biomass is negative in the absence of wolf but neutral in the presence of wolf (Figure 2).

### 3.2 Effect Re‐Establishing Wolf on Scavenger Dynamics

Our simulations showed a general positive trend in scavenger biomass in the presence of wolf (Figure 2). In the absence of boar, we found that wolf could only maintain their presence when the hunting target was high (so when there was little hunting) (Appendix S2: Figure S2.4). In the presence of boar, wolf could maintain their presence with both high and medium hunting targets (Appendix S2: Figure S2.4). In the scenarios where wolf could maintain their presence, we observed more fluctuations in the scavenger biomass around a stable equilibrium (Figure 2), which followed fluctuations in population dynamics of deer and boar (Appendix S2: Figures S2.1 and S2.2). This again is due to general predator prey dynamics, since the fluctuations in biomass of deer and boar followed the fluctuations of wolf biomass and vice versa (Appendix S2: Figures S2.1– S2.4).

### 3.3 Effect of Human Hunting Strategies on Scavenger Dynamics

The hunting target had, via the populations of deer and boar (Appendix S2: Figures S2.1 and  S2.2), a huge effect on scavenger biomass in general (Figure 2). The lower the biomass of deer and boar, the higher the biomass of scavengers, resulting from decreasing competition for vegetation resources. We observed that more hunting resulted in less deer and boar (Appendix S2: Figures S2.1 and S2.2), which subsequently resulted in higher biomass of scavengers (Figure 2).

In the presence of both boar and wolf, medium and high hunting targets caused the same scavenger biomass (Figure 2). The higher the hunting target, the more the wolf took over from humans in killing deer and boar. This often resulted in deer and boar populations below the hunting target in this scenario, meaning that there was no human hunting needed in this scenario to maintain deer and boar population targets (Appendix S2: Figures S2.1 and S2.2). This, in turn, resulted in the same scavenger biomass (Figure 2), although population dynamics fluctuated more when the wolf dominated the hunting.

We found that the fraction of carrion left behind by hunters was only important for scavenger biomass when wolf was absent but boar present (Figure 2). The more carrion that was left behind by hunters, the higher the scavenger biomass (Figure 2). The reason that the extra growth scavengers gained from carrion was only important in this scenario is again due to competition for vegetation resources between scavengers, deer and boar. The vegetation resources were more limited in this scenario than in the three others (Appendix S2: Figure S2.3), and therefore higher fractions of carrion left behind by hunters, actually also resulted in lower populations of deer and boar in this scenario due to competition for vegetation resources with scavengers (Appendix S2: Figures S2.1 and S2.2).

### 3.4 Main Resource for Scavengers

**FIGURE 3:**
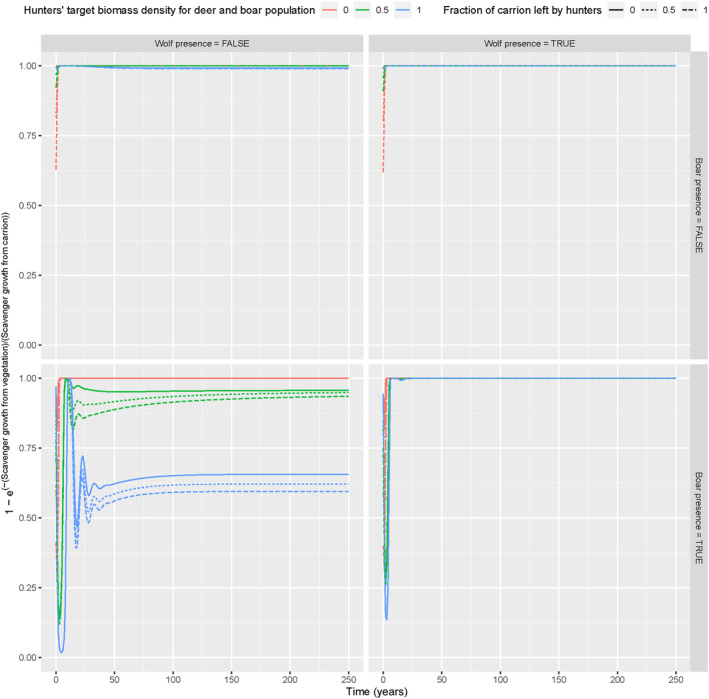
Scavenger growth from vegetation versus scavenger growth from carrion ODE model simulations (*y*‐axis, transformed from [0, ∞] to [0, 1] range) over time (*x*‐axis), with boar (horizontal panels) and wolf present/absent (vertical panels), for different hunting target values (line colours) and fractions of carrion left by hunters (line types).

To assess the main resource for scavengers under different scenarios, we first checked the importance of vegetation versus carrion for the growth of scavenger biomass. Overall, we found that vegetation resources caused way more growth of scavenger biomass compared to carrion (Figure 3). The only exception was when boar was present but wolf absent. Here, the scenarios with high and medium hunting targets resulted in more competition for vegetation resources and simultaneously for more deer and boar biomass that became available as carrion (Figure 3; Appendix S3: Figure S3.5). For that reason, carrion became more important in these scenarios (Figure 3). The sensitivity analysis of the conversion factor of vegetation resources for scavengers indicated that competition for vegetation resources was still a dominant process, rather than the availability of carrion in general, in determining the biomass of scavengers (Appendix S3: Figures S3.1–S3.3).

When wolf was present but boar absent, the available carrion comes either from hunting or from predation (Appendix S2: Figure S2.5). The lower the hunting target, the more carrion was relatively obtained from hunting (Appendix S2: Figure S2.5). In the presence of boar, the fraction of carrion left behind by hunters matters in the case of medium hunting target (Appendix S2: Figure S2.5). In this scenario, we observed that higher fractions of carrion left behind by hunters, the larger the fraction of carrion that is originated from hunting.

**FIGURE 4:**
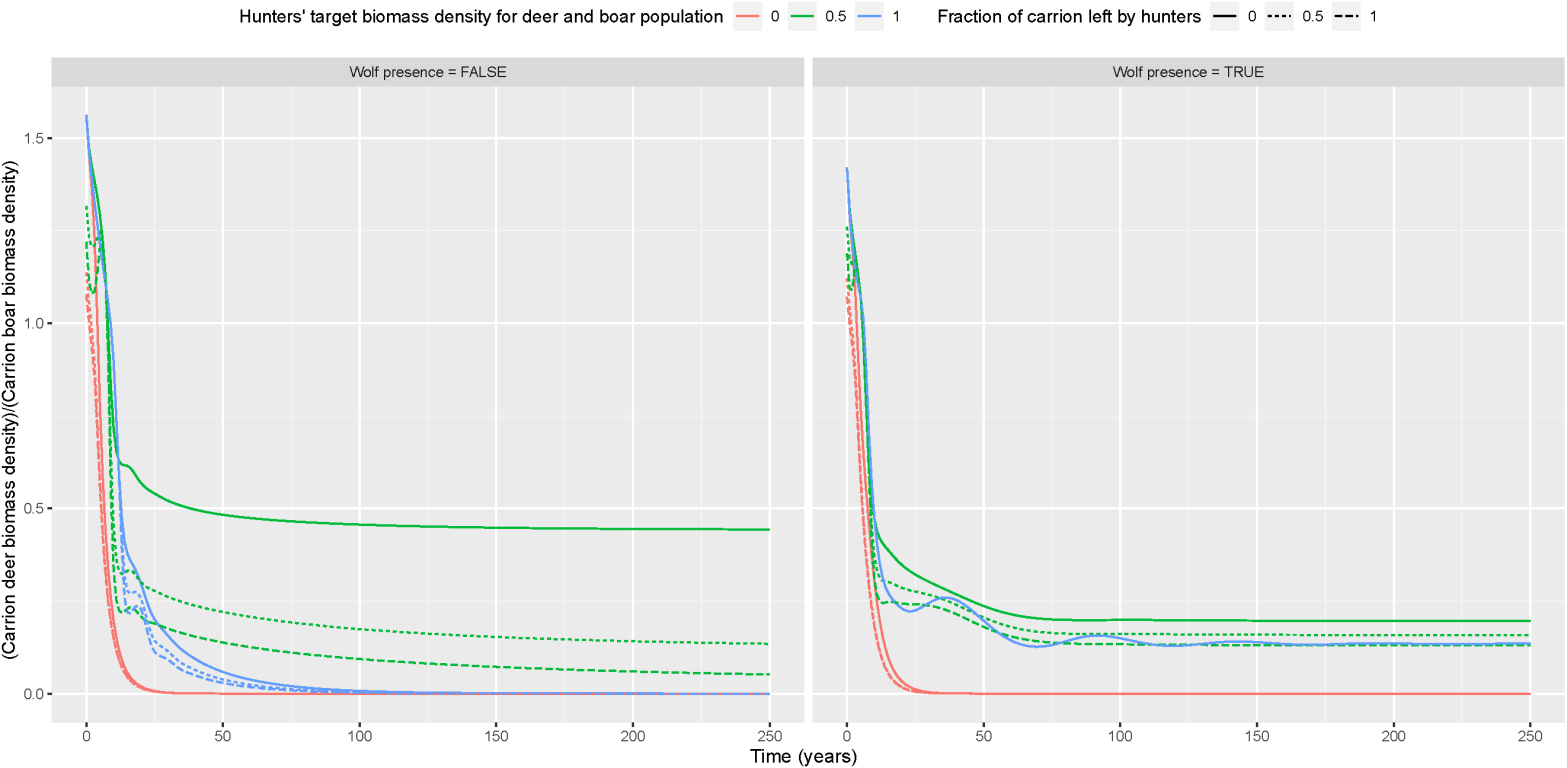
Deer carrion versus boar carrion biomass density ODE model simulations (*y*‐axis) over time (*x*‐axis), with wolf present/absent (panels), for different hunting target values (line colours) and fractions of carrion left by hunters (line types).

In the presence of boar, we found that there was always more boar carrion than deer carrion available (Figure 4). This is because, in general, boar biomass was always higher than deer biomass in our simulations (Appendix S2: Figures S2.1 and S2.2). With a medium hunting target and in the absence of wolf, deer was not outcompeted by boar and scavengers (Appendix S2: Figures S2.1–S2.4). Also, deer was not outcompeted in the presence of wolf, but only when the hunting target was zero (Appendix S2: Figure S2.1). Only in the scenarios with medium hunting target, the fraction of carrion left behind by hunters influenced the fraction of deer versus boar carrion (Figure 4).

## 4 Discussion

In this study, we examined how different human hunting strategies, combined with the presence or absence of boar and wolf, influenced the dynamics of scavenger biomass in a system with only facultative scavengers. We did not aim to create fully realistic scenarios of specific existing natural systems, but intended to create a mathematical model to improve our theoretical understanding of all the interacting processes that are involved. Given the nature of a simulation study, we made many assumptions to simplify reality to obtain generalisable conclusions. These assumptions included that the wolves' diet was exclusively based on deer and boar predation, that there was only one shared vegetation resource for all populations, that wild boar did not scavenge on conspecifics, no scavenging by wolves, no human prosecution of wolves, and that the populations are limited by food (instead of space). Regardless of these assumptions, we found some patterns that provided new insights into the population dynamics of facultative scavengers when wolves and/or boar are re‐establishing under different human hunting strategies.

A key conclusion of our simulations is that carrion was not the most important resource in determining the biomass growth of facultative scavengers (Figure 3). These facultative scavengers are flexible in their diet and behaviour and can therefore adapt to local circumstances (Díaz‐Ruiz et al. 2013; Rooney and Montgomery 2013; Papakosta et al. 2014; Jain et al. 2022). As a result, carrion is not equally consumed among and within ecosystems and different local scavenger guilds, which results in high variability of the carrion decomposition process in general (Newsome et al. 2021; Wenting, Rinzema, and van Langevelde 2022; Wenting et al. 2024; Vandersteen et al. 2023). This implies that carrion is an ephemeral resource for facultative scavengers, which supplements their diet and behaviour but does not necessarily determine it (Wilson and Wolkovich 2011; Barton et al. 2013), which is in line with our results. Moreover, the presence of wolves also has indirect effects by changing intraguild dynamics between large and small prey species (Ripple and Beschta 2004; Jędrzejewski et al. 2012), ultimately changing dynamics among facultative scavenger guilds (Wikenros et al. 2013) and vegetation resources (Jędrzejewski et al. 2012; Kuijper et al. 2013).

Due to the direct competition for vegetation resources in our model by deer and boar with scavengers, we assumed that the competitive release hypothesis (Ketterson and Nolan Jr 1976; Le Bagousse‐Pinguet, Gross, and Straile 2012) applies to our study system. As such, a lower population of one group often positively impacts the populations of other groups (Berg et al. 2019; Van Moorter et al. 2021). This has been demonstrated for the European ecosystems where wolves are present (Chapman et al. 2011), which is reflected in our results (Appendix S2: Figures S2.1–S2.4).

The presence of wolf had an overall positive effect on the scavenger population and could take over the role of human hunting in controlling ungulate populations under some conditions (Figure 2). In our model, wolf was fully dependent on predation on deer and boar. It can supplement its diet with other resources, including livestock (Janeiro‐Otero et al. 2020) and carrion (Petroelje et al. 2019; Wirsing and Newsome 2021). Carrion consumption by wolves is extensively documented in some ecosystems (Mateo‐Tomás et al. 2015). In temperate ecosystems, on which our simulations were based, it has only been proven in areas where wolves were re‐established for multiple years, or where they were never extinct (Jędrzejewski et al. 2002; Selva 2004; Selva and Fortuna 2007). In other areas, where wolves recently re‐established, evidence is only anecdotical or absent. Thus, it is unknown whether recently re‐established wolves scavenge substantially or change their scavenging habits over time. Based on this, we decided to simplify the model by only focusing on scavenging by facultative scavengers and hence not to include scavenging behaviour of wolves.

Depending on the local circumstances, including the presence of large carnivores (that can induce fear), facultative scavengers establish a specific way of scavenging behaviour (Selva et al. 2005; Pereira, Owen‐Smith, and Moleón 2014; Kane et al. 2017). For example, the willingness of species to forage in open areas decreases with increasing predation pressure (Allen et al. 2015), in line with the ecology of fear (Haswell et al. 2018, 2020; Gaynor et al. 2021; Ramirez et al. 2024). This, in turn, might reduce the potential effects of habitat type on scavenging behaviour in general, meaning that scavengers might forage more in open landscapes instead of forests only, and vice versa (Wenting et al. 2024). We suppose that facultative scavengers, due to their adaptable nature, eventually adapt their scavenging habits when large carnivores re‐establish. However, the question is about the speed at which they will adapt their behaviour. This might cause some iterations in scavenger dynamics when wolves re‐establish, until scavengers have adapted their behaviour to the wolves' presence. However, the ultimate consequences are unclear and hard to predict, especially in human‐dominated landscapes (Hebblewhite et al. 2005; Dorresteijn et al. 2015).

Boar outcompeted deer in the scenarios with low hunting pressure and without wolves (Appendix S2: Figures S2.1 and S2.2). This is because we assumed boar to be more efficient in exploiting vegetation resources than deer, i.e., boar had a higher conversion factor of vegetation resources than deer (Table 1), mainly due to their higher reproductive rate (Appendix S1). For simplicity, we used only one vegetation resource for all species. Consequently, boar and deer competed directly for exactly the same resource. This is not realistic due to niche differentiation among those boar and deer species (Gebert and Verheyden‐Tixier 2001; Ballari and Barrios‐García 2014; Mikulka et al. 2018; Spitzer et al. 2020). The same applies to facultative scavengers; although they are predominantly omnivores, e.g., Red fox and European badger, that contain plant‐based resources in their diet, the vegetation they consume do not fully overlap with deer and boar (Castañeda et al. 2022; Jain et al. 2022). We assume this simplification to be the main limitation of our model for interpreting our results. However, although in reality the resources of all the species do not fully overlap, it is still reasonable that they do show some overlap. The absolute values of our results do not have any predictive power for reality, but the patterns that we modelled still do, which is exemplified by our sensitivity analyses on the vegetation conversion coefficients by scavengers (Appendix S3: Figures S3.1–S3.3). Therefore, our result that carrion might not be the main resource that determines the biomass growth of facultative scavengers is still valid.

We found that the presence of boar on scavenger biomass was negative when wolf was absent but neutral when wolf was present (Figure 2). However, scavenger biomass does not automatically reflect the functionality of the scavenger community and the potential effects that scavengers can have on ecological processes. Nonetheless, the simulations are in line with the alleged unique role of boars in carrion decomposition (Wenting, Rinzema, and van Langevelde 2022; Wenting et al. 2024). Also, based on our simulations, we expect that the co‐occurrence of both boar and wolf stimulates fundamental ecological processes – e.g., nutrient cycling and restoring biodiversity – the most.

Our simulations with and without boar's presence can be seen as an example of human influences that extend beyond hunting. Both boar and wolf are involved in human‐wildlife conflicts (Massei et al. 2015; Storie and Bell 2017; Kuijper et al. 2019; König et al. 2020). Wolf is, unlike boar, strictly protected by law in the EU, meaning that their presence needs to be tolerated (Trouwborst and Fleurke 2019). Boars are not tolerated everywhere, or their populations are extensively controlled (Thurfjell, Spong, and Ericsson 2013; Massei et al. 2015). Our simulations imply, however, that the coexistence of both boar and wolf would positively influence the scavenger dynamics in general by increasing the overall scavenger biomass densities. Consequently, the co‐existence of both species would, eventually, enhance the overall ecosystem functioning. We consider this as the most noticeable conclusion of our study.

When the hunting target was low, wolf could replace the effects of human hunting by keeping the populations of deer and boar below the hunting target (Appendix S2: Figures S2.1 and S2.2). That implies that human hunting in general should be reconsidered and adapted to re‐establishing wolf populations. This has not only ecological benefits, as our model implies (Figure 2), but would also reduce human‐wildlife conflicts since it has been widely documented that established wolves prefer wild prey over livestock (Meriggi and Lovari 1996; Sidorovich, Tikhomirova, and Jędrzejewska 2003; Ferretti et al. 2019).

In conclusion, our model indicates that population dynamics of facultative scavengers are not mainly driven by the availability of carrion but rather by the presence of and competition for vegetation and other resources. The co‐occurrence of boar and wolf can have positive effects on scavengers' population dynamics. Their population dynamics showed more fluctuations as human hunting, to control deer and boar densities, was taken over by wolves. Although this is in line with well‐documented natural predator–prey interactions (Wangersky and Cunningham 1957; Mougi and Iwasa 2010), it highlights the importance of changing the human hunting strategy in accordance with wolves' re‐establishment.

## Author Contributions

**Elke Wenting:** conceptualization (lead), formal analysis (equal), methodology (equal), software (supporting), visualization (equal), writing – original draft (equal), writing – review and editing (equal). **Jasper A. J. Eikelboom:** conceptualization (supporting), formal analysis (equal), methodology (equal), software (lead), visualization (equal), writing – original draft (equal), writing – review and editing (equal). **Henk Siepel:** conceptualization (supporting), methodology (supporting), writing – original draft (supporting). **Femke Broekhuis:** conceptualization (supporting), writing – original draft (supporting). **Frank van Langevelde:** conceptualization (supporting), methodology (supporting), writing – original draft (supporting).

## Conflicts of Interest

The authors declare no conflicts of interest.

### Open Research Badges

This article has earned an Open Data badge for making publicly available the digitally‐shareable data necessary to reproduce the reported results. The data is available at https://doi.org/10.4121/a5a040e7‐de45‐4d60‐9ac4‐eec4e826aa8.

## Supporting information


Appendix S1.

Appendix S2.

Appendix S3.

## Data Availability

The complete R script of the ODE model, including all sensitivity analyses to produce the manuscript figures, is available via: https://doi.org/10.4121/a5a040e7‐de45‐4d60‐9ac4‐eec4e826aa85.
