# Supplementary material for: Influence of Human Hunting Strategies and Large Carnivore Presence on Population Dynamics of European Facultative Scavengers
Source: Ecol Evol. 2024 Nov 4;14(11):e70424. doi: 10.1002/ece3.70424 (PMC11534445; doi:10.1002/ece3.70424)
Supplement: Supplementary file 1 — Appendix S1. [file ECE3-14-e70424-s002.docx]

Table S1.1 Calculation conversion coefficient deer.

| **Factor** | **Unit** | **Value** | **Remarks / explanation** |
| --- | --- | --- | --- |
| Intake |  |  |  |
| - Interbirth interval (1) | y | 1 | based on Flajšman et al. (2017) |
| - Food requirement (2) | kg/y/ind | 986 | on average 2.7 kg per day (Mulley 2002) |
| - Fraction of reproductive females (3) | ind/ind | 0.4 |  |
| *Total intake* | kg/ind | 2465 | (1) * (2) / (3) |
| Output |  |  |  |
| - Weight of young after lactation (4) | kg/ind | 42.24 | based on Moore et al. (1988) |
| - Average litter size per female (5) | ind/ind | 1 | based on Flajšman et al. (2017) |
| *Total output* | kg/ind | 42.24 | (4) * (5) |
| Conversion coefficient |  | 0.017 | Total output / Total intake |

Table S1.2 Calculation conversion coefficient boar.

| **Factor** | **Unit** | **Value** | **Remarks / explanation** |
| --- | --- | --- | --- |
| Intake |  |  |  |
| - Interbirth interval (1) | y | 0.75 | Chinn et al. (2022), on average 1.5 litter per year for simplicity |
| - Food requirement (2) | kg/y/ind | 1209.4 | Nagy (2021), Treyer et al. (2012) |
| - Fraction of reproductive females (3) | ind/ind | 0.4 |  |
| *Total intake* | kg/ind | 2267.7 | (1) * (2) / (3) |
| Output |  |  |  |
| - Weight of young after lactation (4) | kg/ind | 23 | Treyer et al. (2012) |
| - Average litter size per female (5) | ind/ind | 5.68 | Gethöffer et al. (2007) |
| *Total output* | kg/ind | 130.64 | (4) * (5) |
| Conversion coefficient (i) |  | 0.058 | Total output / Total intake |
| Portion carrion in diet (ii) |  | 0.16 |  |
| Digestibility of vegetation (iii) |  | 0.8 | animal matter 100%, plant matter 80% (Sá et al. 2020) |
| Conversion coefficient scaled (vi) |  | 0.069 | (i) / ( (ii) + ( 1 – (ii) ) * (iii) ) |
| Conversion coefficient vegetation |  | 0.055 | (vi) * (iii) |
| Conversion coefficient animal matter |  | 0.069 | (vi) |

Table S1.3 Calculation conversion coefficient wolf.

| **Factor** | **Unit** | **Value** | **Remarks / explanation** |
| --- | --- | --- | --- |
| Intake |  |  |  |
| - Interbirth interval (1) | y | 1 |  |
| - Food requirement (2) | kg/y/ind | 1642.5 | 4.5 kg per day * 365 days (Jędrzejewski et al. 2002) |
| - Fraction of reproductive females (3) | ind/ind | 0.4 |  |
| *Total intake* | kg/ind | 2465 | (1) * (2) / (3) |
| Output |  |  |  |
| - Weight of young after lactation (4) | kg/ind | 25 | based on Jędrzejewski et al. (2002) |
| - Average litter size per female (5) | ind/ind | 6.25 | average, based on Sidorovich et al. (2007) |
| *Total output* | kg/ind | 156.25 | (4) * (5) |
| Conversion coefficient |  | 0.038 | Total output / Total intake |

Table S1.4 Calculation conversion coefficient scavengers.

| **Factor** | **Value** | **Remarks / explanation** |
| --- | --- | --- |
| *Vegetation* |  |  |
| - Conversion coefficient deer (1) | 0.017 |  |
| - Conversion coefficient boar (veg) (2) | 0.055 |  |
| Conversion coefficient vegetation | 0.036 | ( (1) + (2) ) / 2 |
| *Animal matter* |  |  |
| - Conversion coefficient wolf (3) | 0.038 |  |
| - Conversion coefficient boar (animal) (4) | 0.069 |  |
| Conversion coefficient animal matter | 0.054 | ( (3) + (4) ) / 2 |
