# Supplementary material for: Influence of Human Hunting Strategies and Large Carnivore Presence on Population Dynamics of European Facultative Scavengers
Source: Ecol Evol. 2024 Nov 4;14(11):e70424. doi: 10.1002/ece3.70424 (PMC11534445; doi:10.1002/ece3.70424)
Supplement: Supplementary file 2 — Appendix S2. [file ECE3-14-e70424-s001.docx]

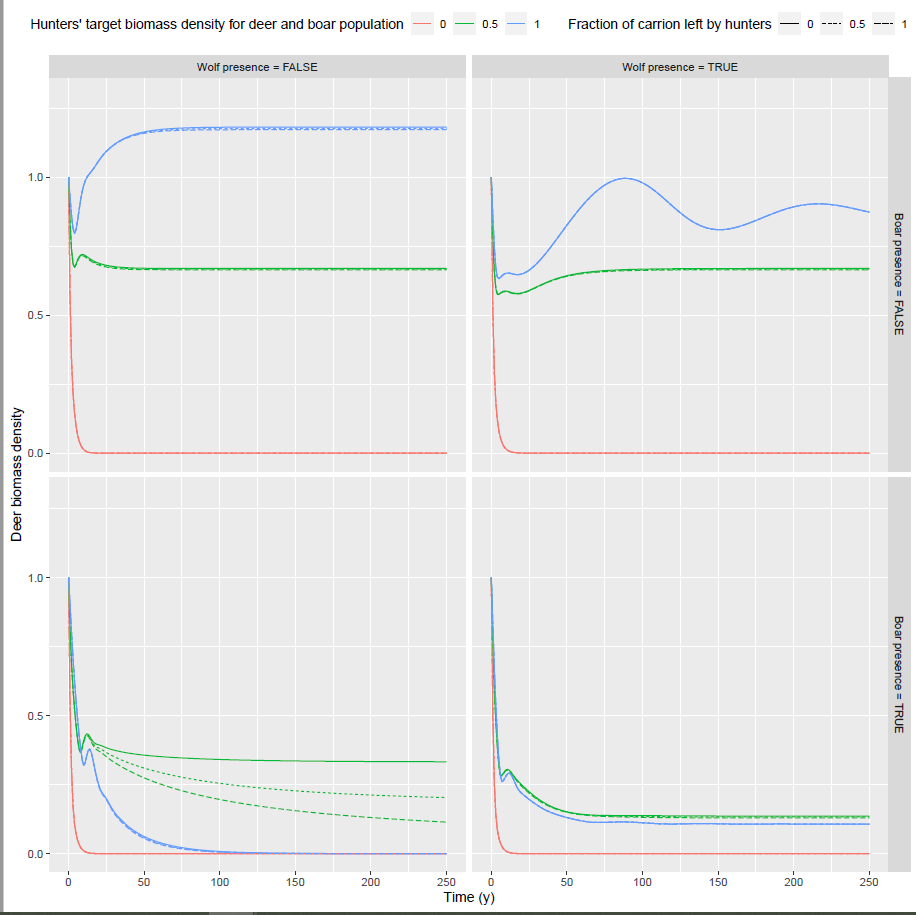


Fig. S2.1 Deer biomass density ODE model simulations (y-axis) over time (x-axis), with boar (horizontal panels) and wolf present/absent (vertical panels), for different hunting target values (line colours) and fractions of carrion left by hunters (line types).


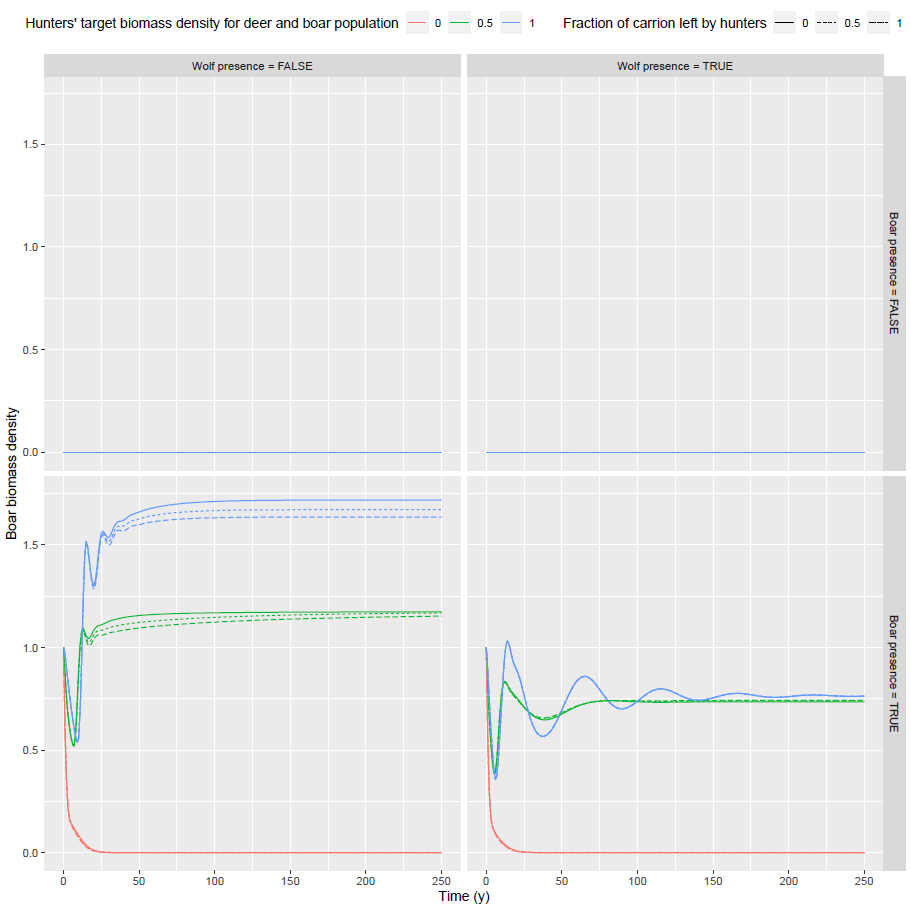


Fig. S2.2 Boar biomass density ODE model simulations (y-axis) over time (x-axis), with boar (horizontal panels) and wolf present/absent (vertical panels), for different hunting target values (line colours) and fractions of carrion left by hunters (line types).


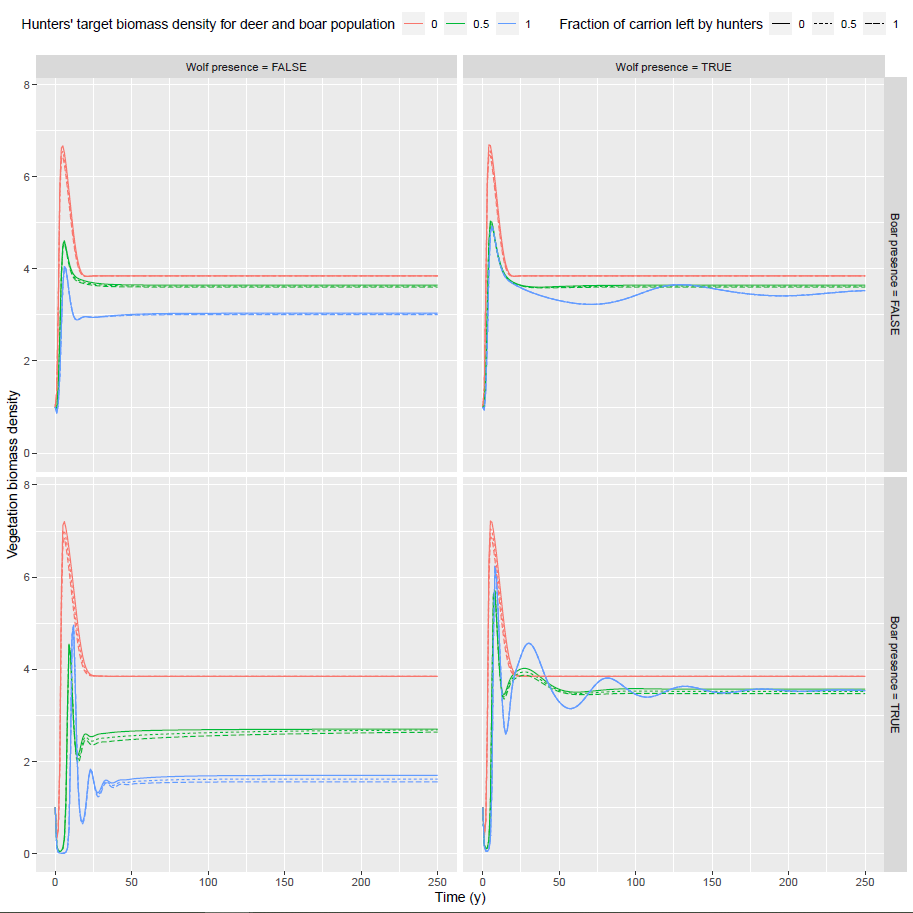


Fig. S2.3 Vegetation biomass density ODE model simulations (y-axis) over time (x-axis), with boar (horizontal panels) and wolf present/absent (vertical panels), for different hunting target values (line colours) and fractions of carrion left by hunters (line types).


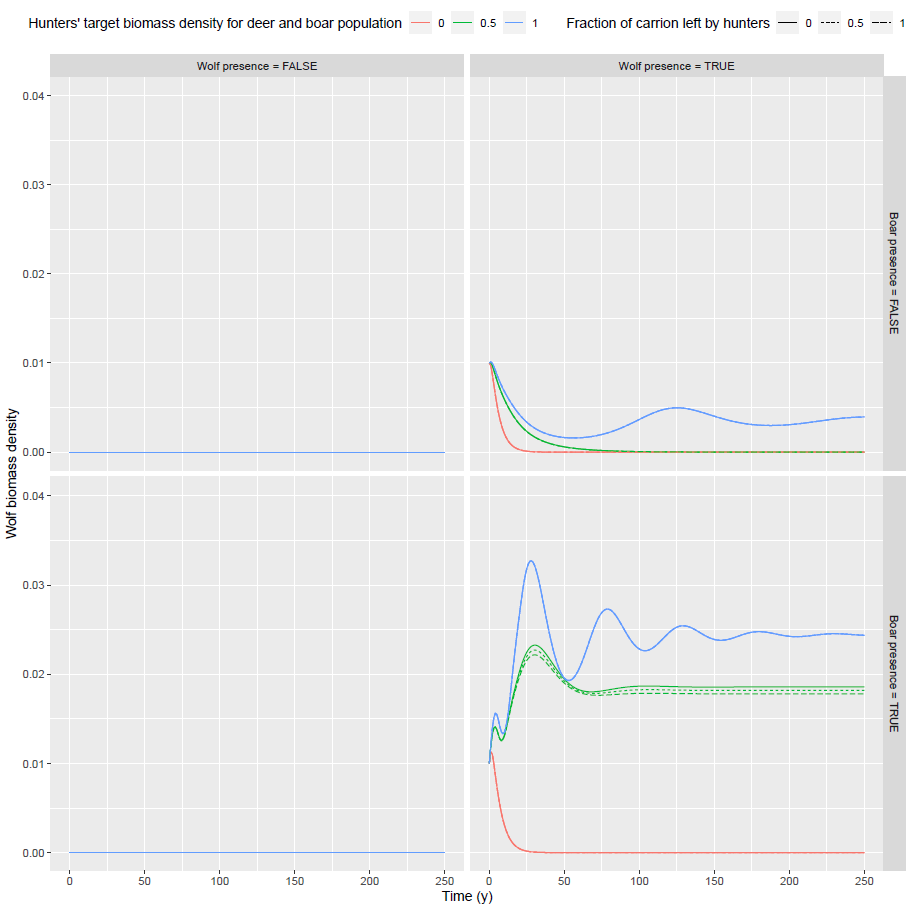


Fig. S2.4 Wolf biomass density ODE model simulations (y-axis) over time (x-axis), with boar (horizontal panels) and wolf present/absent (vertical panels), for different hunting target values (line colours) and fractions of carrion left by hunters (line types).


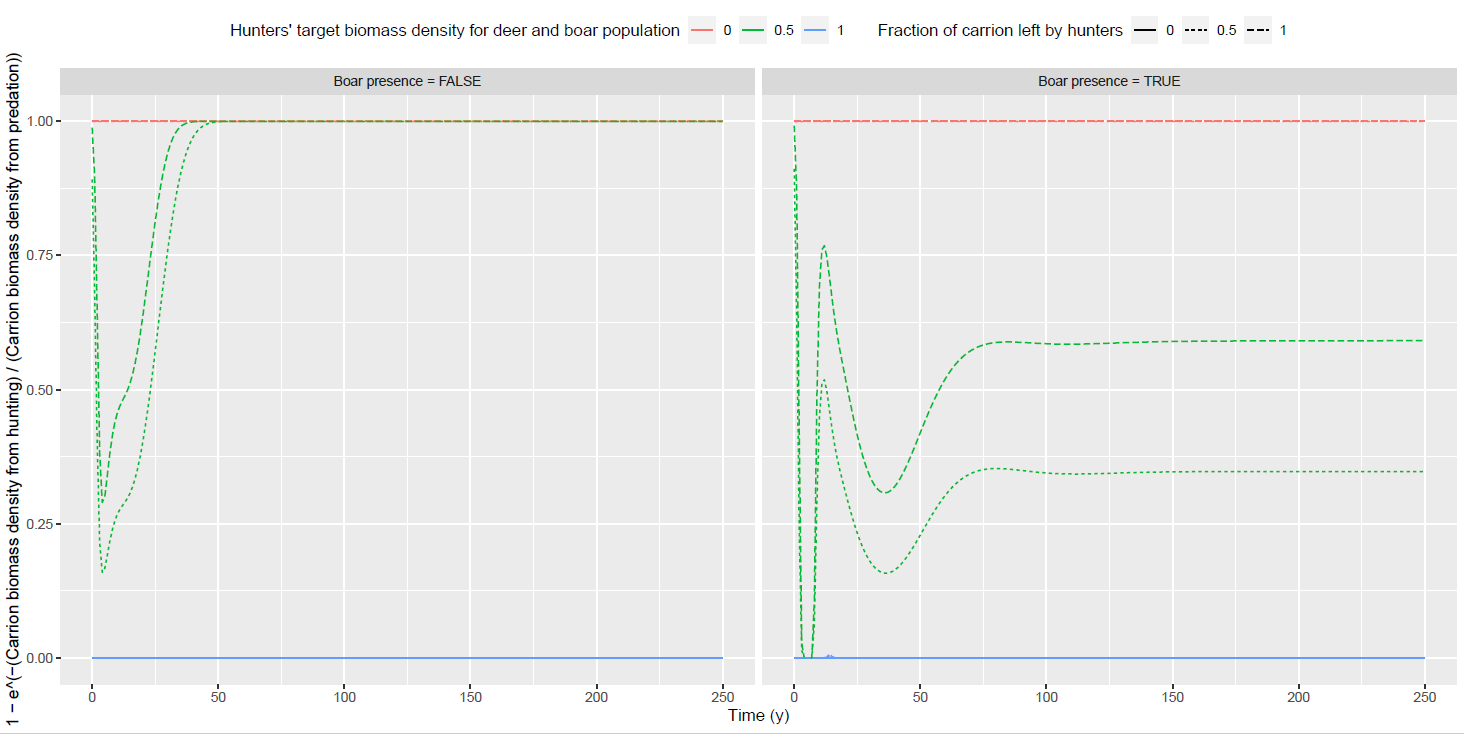


Fig. S2.5 Scavenger growth from predated carrion versus growth from carrion left behind from hunting ODE model simulations (y-axis, transformed from [0,∞] to [0,1] range) over time (x-axis), with boar present/absent (vertical panels), for different hunting target values (line colours) and fractions of carrion left by hunters (line types).
