# Supplementary material for: Influence of Human Hunting Strategies and Large Carnivore Presence on Population Dynamics of European Facultative Scavengers
Source: Ecol Evol. 2024 Nov 4;14(11):e70424. doi: 10.1002/ece3.70424 (PMC11534445; doi:10.1002/ece3.70424)
Supplement: Supplementary file 3 — Appendix S3. [file ECE3-14-e70424-s003.docx]

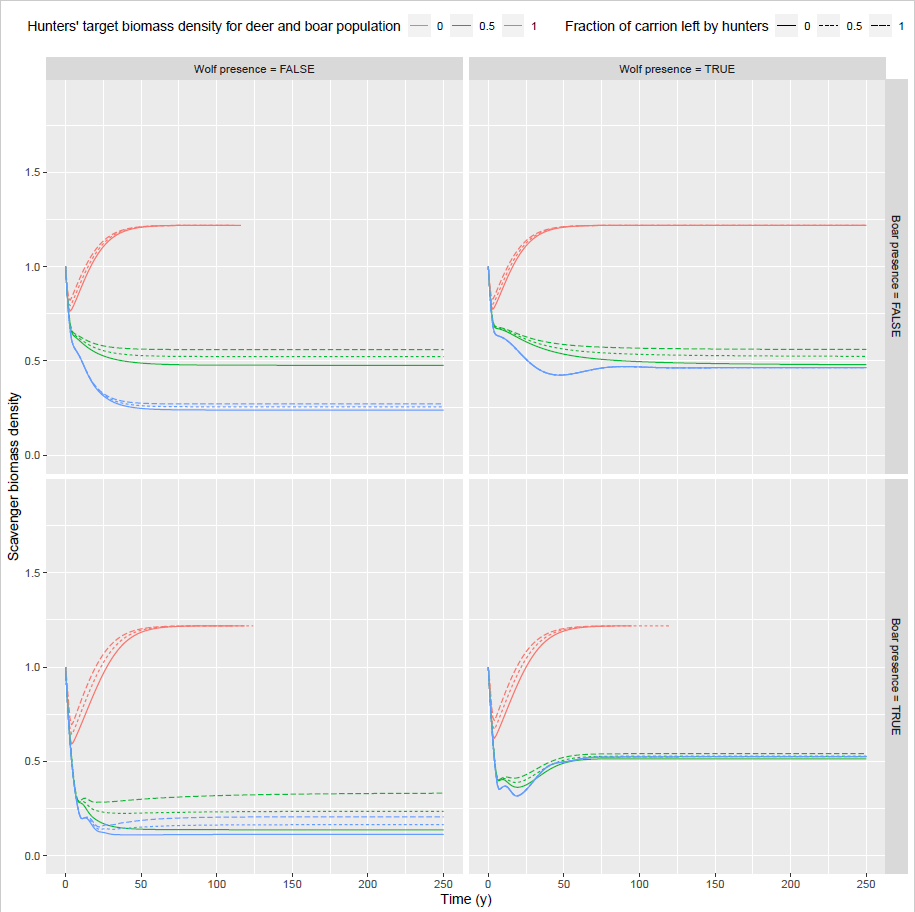


Fig. S3.1 Scavenger biomass density ODE model simulations (y-axis) over time (x-axis), for low vegetation conversion factor, with boar (horizontal panels) and wolf present/absent (vertical panels), for different hunting target values (line colours) and fractions of carrion left by hunters (line types).


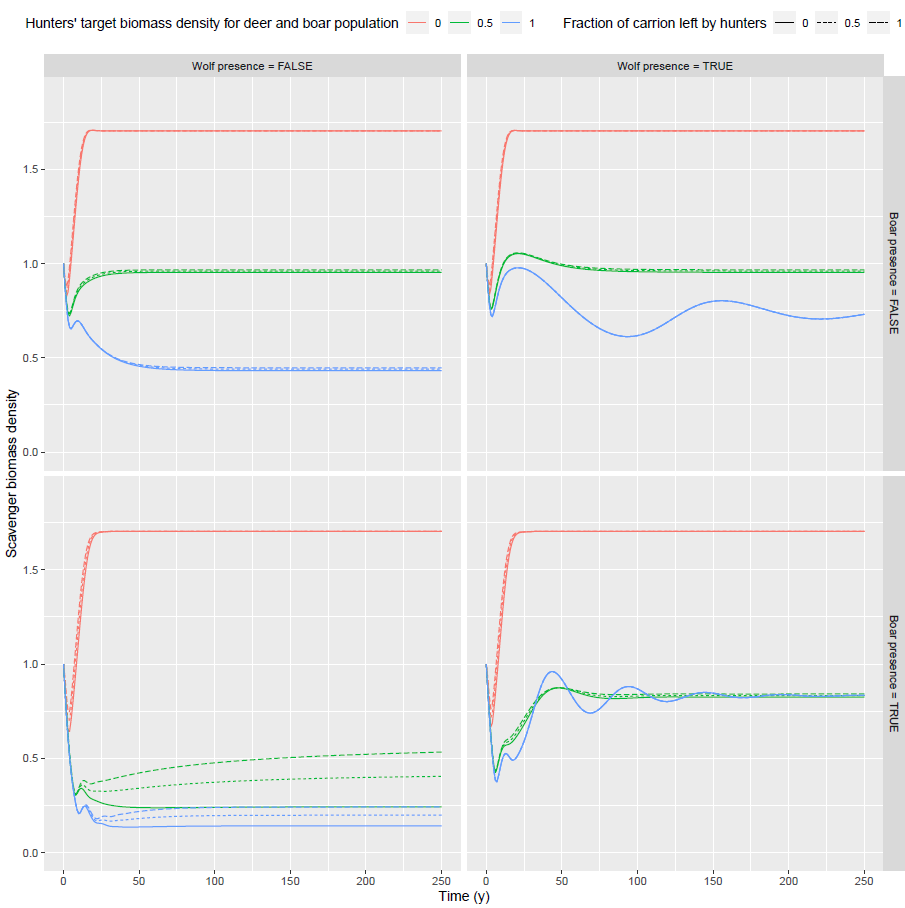


Fig. S3.2 Scavenger biomass density ODE model simulations (y-axis) over time (x-axis), for medium vegetation conversion factor, with boar (horizontal panels) and wolf present/absent (vertical panels), for different hunting target values (line colours) and fractions of carrion left by hunters (line types).


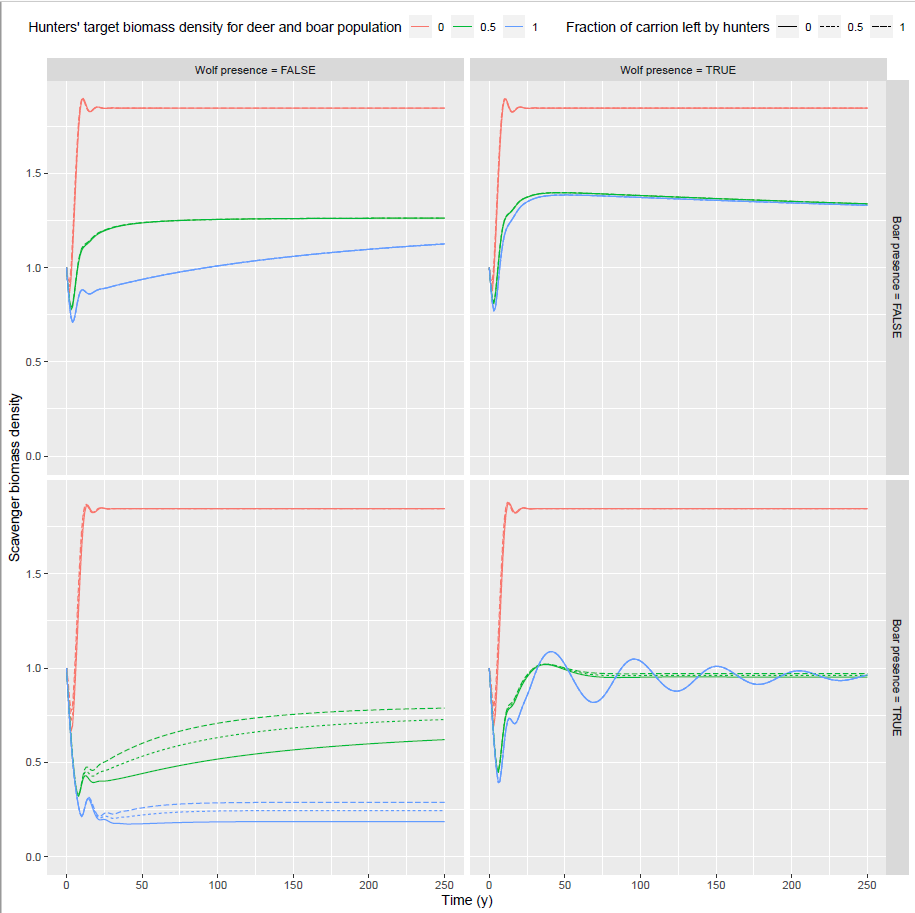


Fig. S3.3 Scavenger biomass density ODE model simulations (y-axis) over time (x-axis), for high vegetation conversion factor, with boar (horizontal panels) and wolf present/absent (vertical panels), for different hunting target values (line colours) and fractions of carrion left by hunters (line types).


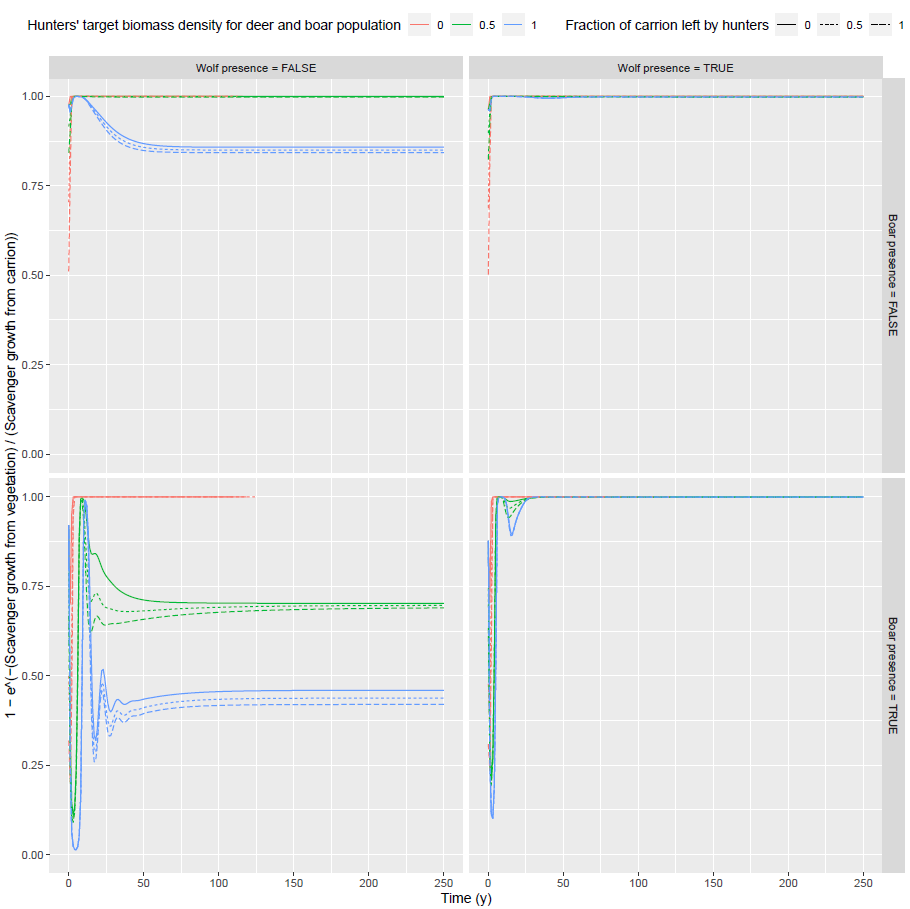


Fig. S3.4 Scavenger growth from vegetation versus scavenger growth from carrion ODE model simulations (y-axis, transformed from [0,∞] to [0,1] range) over time (x-axis), for low vegetation conversion factor of scavengers, with boar (horizontal panels) and wolf present/absent (vertical panels), for different hunting target values (line colours) and fractions of carrion left by hunters (line types).


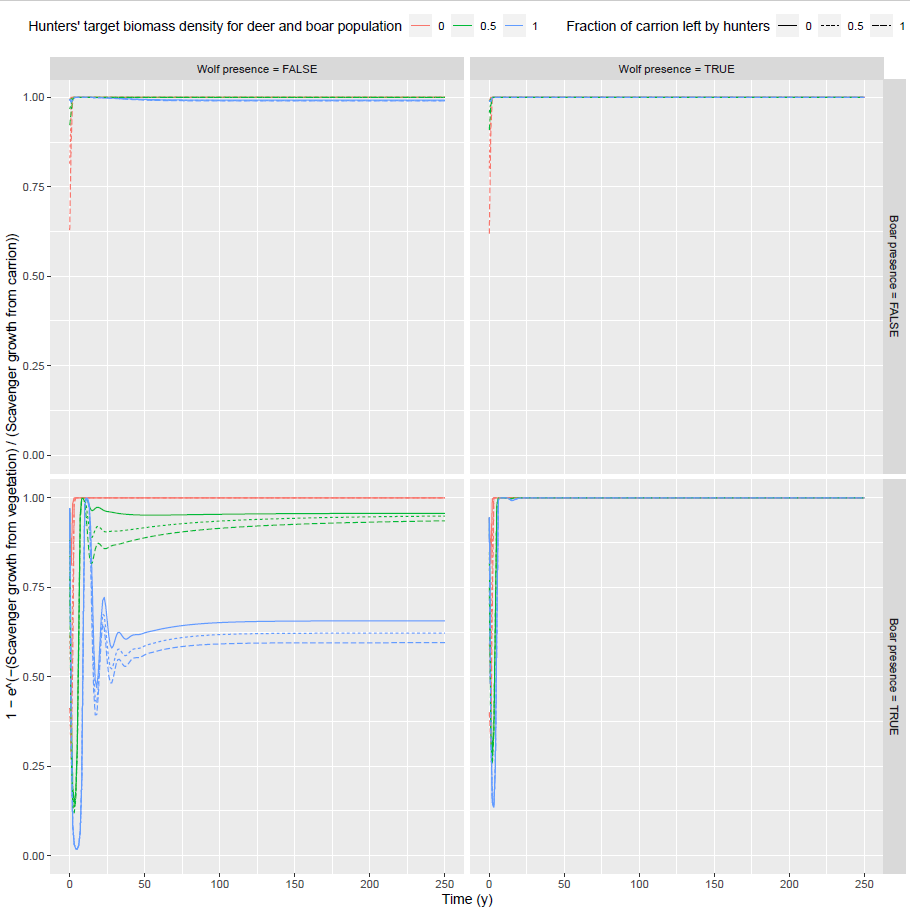


Fig. S3.5 Scavenger growth from vegetation versus scavenger growth from carrion ODE model simulations (y-axis, transformed from [0,∞] to [0,1] range) over time (x-axis), for medium vegetation conversion factor of scavengers, with boar (horizontal panels) and wolf present/absent (vertical panels), for different hunting target values (line colours) and fractions of carrion left by hunters (line types).


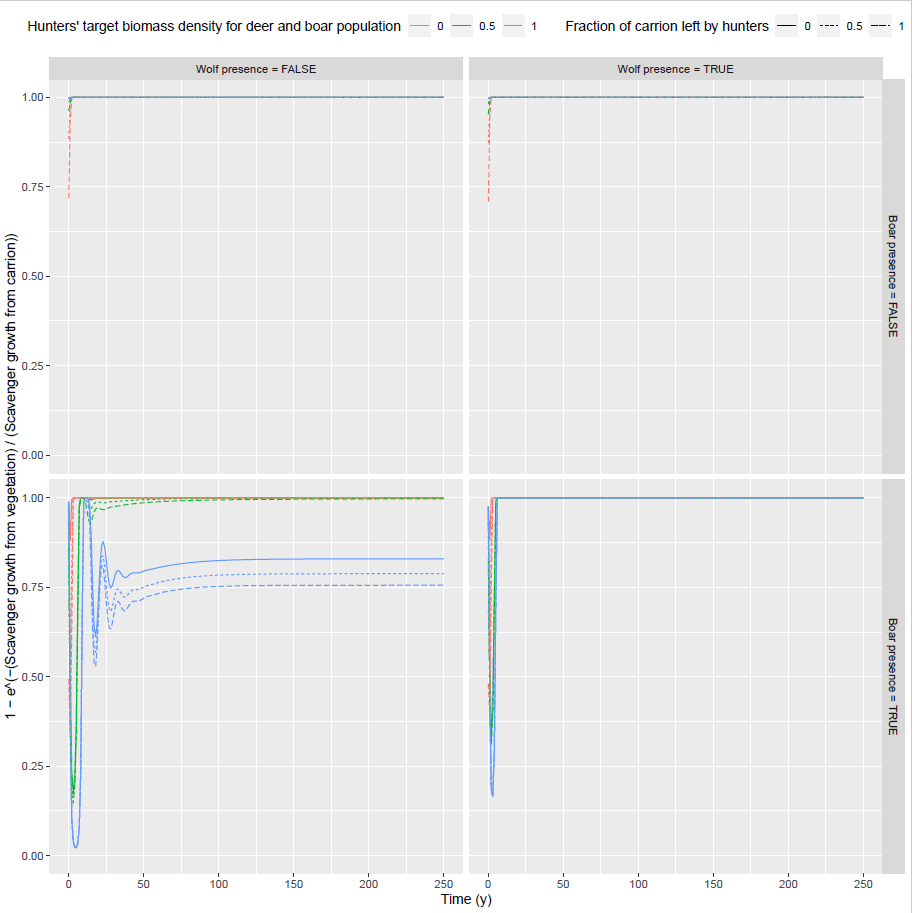


Fig. S3.6 Scavenger growth from vegetation versus scavenger growth from carrion ODE model simulations (y-axis, transformed from [0,∞] to [0,1] range) over time (x-axis), for high vegetation conversion factor of scavengers, with boar (horizontal panels) and wolf present/absent (vertical panels), for different hunting target values (line colours) and fractions of carrion left by hunters (line types).
